# Tissue Homogenate VEGF Levels in Malignant Salivary Gland Tumors vs. Controls: An Exploratory Pilot Study

**DOI:** 10.3390/jcm15124557

**Published:** 2026-06-12

**Authors:** Wojciech Domka, Maciej Misiołek, Tomasz Kubrak, Angelika Myśliwiec, Agnieszka Przygórzewska, Dorota Bartusik-Aebisher, David Aebisher

**Affiliations:** 1 Department of Otolaryngology, Collegium Medicum, Faculty of Medicine, University of Rzeszów, 35-959 Rzeszów, Poland; 2 Department of Otorhinolaryngology and Oncological Laryngology in Zabrze, Medical University of Silesia, 40-055 Katowice, Poland; maciej.misiolek@sum.edu.pl; 3 Department of Biochemistry and General Chemistry, Collegium Medicum, Faculty of Medicine, University of Rzeszów, 35-959 Rzeszów, Poland; tkubrak@ur.edu.pl (T.K.); amysliwiec@ur.edu.pl (A.M.); dbartusikaebisher@ur.edu.pl (D.B.-A.); 4 Doctoral School of University of Rzeszów, Collegium Medicum, Faculty of Medicine, University of Rzeszów, 35-959 Rzeszów, Poland; ap117623@stud.ur.edu.pl; 5 Department of Photomedicine and Physical Chemistry, Faculty of Medicine, University of Rzeszów, 35-959 Rzeszów, Poland

**Keywords:** salivary gland tumors, vascular endothelial growth factor, angiogenesis, pilot study

## Abstract

**Background/Objectives:** Tumors of the salivary glands represent a rare and heterogeneous group of tumors, the diagnosis and treatment of which present significant clinical challenges. One of the key processes affecting the progression of these tumors is angiogenesis, in which vascular endothelial growth factor (VEGF) and its receptors play a fundamental role. The aim of the present study was to assess differences in VEGF levels in salivary gland tumor tissue compared with normal salivary gland tissue. **Methods:** Salivary gland tissue samples were obtained by surgical resection. Thirteen patients were included in the study, nine with malignant lesions and four patients with normal salivary gland tissue as controls. After tissue homogenization, VEGF concentration was analyzed by a chemiluminescent ELISA (Human VEGF QuantiGlo ELISA Kit, QVE00B; R&D Systems, Minneapolis, MN, USA). **Results:** VEGF concentrations in salivary gland tumor tissue homogenate supernatants showed a higher central tendency than controls, but the between-group difference did not reach statistical significance (Mann–Whitney U test, *p* = 0.199; Welch’s *t*-test as sensitivity analysis, *p* = 0.102). **Conclusions:** VEGF quantification in salivary gland tissue homogenate supernatants was feasible and showed substantial inter-individual variability with partial overlap between tumors and controls. Although tumors showed higher central tendency, the differences were not statistically significant in this small cohort; therefore, the findings are hypothesis-generating and do not support diagnostic or prognostic claims.

## 1. Introduction

Salivary gland tumors are rare neoplasms, occurring at a rate of approximately 2.5–3.0 cases per 100,000 people per year in Western countries and accounting for about 5% of all head and neck cancers [1]. Most tumors arise in the parotid glands, whereas submandibular, minor, and sublingual salivary glands are affected less frequently [1,2]. These tumors are characterized by considerable histopathological diversity and variable clinical behavior, which makes their diagnosis, prognosis, and treatment challenging [3,4].

According to the WHO classification, salivary gland tumors include a broad spectrum of benign and malignant epithelial neoplasms. Benign tumors include mainly pleomorphic adenoma and Warthin tumor, whereas malignant tumors include, among others, mucoepidermoid carcinoma, adenoid cystic carcinoma, acinic cell carcinoma, salivary duct carcinoma, and carcinoma ex pleomorphic adenoma [2,3,4].

One of the key processes involved in tumor progression is angiogenesis, which supports tumor growth, local invasion, and metastatic spread [5,6,7]. Vascular endothelial growth factor (VEGF) is one of the main regulators of this process and may have prognostic significance in salivary gland tumors [5,6]. The VEGF family includes VEGF-A, VEGF-B, VEGF-C, VEGF-D, and placental growth factor (PlGF), which are involved in angiogenesis, lymphangiogenesis, inflammatory responses, oxidative stress, and metabolic regulation [6,8].

Among these factors, VEGF-A is the most extensively studied mediator of tumor angiogenesis [5,6]. Its expression is strongly induced by tissue hypoxia through hypoxia-inducible transcription factors, which activate VEGF-A gene transcription and promote neovascularization mainly via VEGFR-2 [5,6,7]. Other VEGF family members also contribute to tumor biology: VEGF-B may support cell survival and tumor progression, VEGF-C and VEGF-D are involved in lymphangiogenesis and lymphatic metastasis, while PlGF mainly participates in pathological angiogenesis and inflammatory conditions [6,8].

Recent studies indicate that VEGF signaling should not be interpreted as a marker of blood vessel formation only. VEGF-related pathways are also associated with endothelial cell metabolism, tumor microenvironment regulation, immune cell recruitment, immunosuppression, and resistance to anticancer therapy [5,6,8,9,10,11,12,13]. These mechanisms suggest that VEGF may influence not only tumor vascularization but also biological aggressiveness and response to treatment.

The use of VEGF as a biomarker in salivary gland tumors remains a promising but still insufficiently established research direction [14,15]. Immunohistochemical studies and meta-analyses suggest that VEGF expression in malignant salivary gland tumors may be associated with adverse clinicopathological features and poorer outcomes, although available data remain heterogeneous [15]. However, the interpretation of VEGF levels is complicated by tumor heterogeneity, the influence of the tumor microenvironment, and the fact that VEGF may also be present in non-neoplastic tissues [3,15].

Therefore, further studies are needed to clarify the significance of VEGF in salivary gland tumors. The purpose of the present study was to evaluate differences in VEGF levels in salivary gland tumor tissue compared with normal salivary gland tissue. We hypothesized that tumor tissues would show higher VEGF levels, reflecting angiogenic activation. Because this study was designed as an exploratory pilot analysis, its aim was also to provide preliminary effect-size estimates for future studies involving larger and clinically stratified cohorts.

## 2. Materials and Methods

### 2.1. Collection of Tissue Samples from Salivary Glands

The study was conducted at the Department of Otolaryngology, Pediatric Otolaryngology and Laryngologic Oncology of University Clinical Hospital No. 1 in Rzeszów. Tissue samples from salivary glands were obtained during surgical resection. Thirteen patients were included in the study: nine patients with malignant salivary gland tumors and four non-oncological controls providing macroscopically normal submandibular gland tissue. Control tissue consisted of macroscopically normal submandibular gland fragments obtained from patients undergoing surgery for non-neoplastic indications. Routine histopathological examination confirmed the absence of neoplastic lesions in control specimens. For patients with salivary gland tumors, the histological type, grade, stage, and tumor location were recorded based on the available clinical and histopathological documentation. Detailed clinicopathological characteristics of the salivary gland tumor samples are provided in Supplementary Table S1.

The study was conducted in accordance with the Declaration of Helsinki and approved by the Bioethics Committee of the District Medical Chamber in Rzeszów (8 August 2019, approval number: 93/B/2019, with an extension of ethical approval validity until 31 December 2024).

### 2.2. Eligibility Criteria

Patients were eligible for inclusion in the tumor group if they underwent surgical resection for a salivary gland tumor and the diagnosis of malignant salivary gland neoplasm was confirmed by routine postoperative histopathological examination. Additional inclusion criteria were the availability of sufficient tissue material for VEGF analysis, complete basic clinicopathological data, and tissue processing according to the study protocol.

Patients were eligible for inclusion in the control group if they underwent surgery for non-oncological indications and provided macroscopically normal submandibular gland tissue. Control specimens were included only when routine histopathological examination confirmed the absence of neoplastic lesions.

Exclusion criteria for both groups included insufficient tissue material, inadequate sample quality, repeated freeze–thaw cycles, incomplete clinicopathological documentation, or tissue processing not consistent with the study protocol. Patients were also excluded if the available tissue showed extensive necrosis or if the control specimen showed histopathological evidence of neoplastic disease. When documented, patients who had received prior radiotherapy or chemotherapy to the head and neck region before tissue collection were also excluded, as these treatments could potentially influence VEGF expression.

### 2.3. Sample Preparation Procedure

Tumor and control salivary gland tissues were snap-frozen in liquid nitrogen and stored at −80 °C until analysis. On the day of analysis, approximately 300–500 mg of tissue was rinsed in ice-cold phosphate-buffered saline (PBS; pH 7.0–7.4) to remove residual blood, blotted dry, weighed, and minced on ice. Tissue was homogenized in ice-cold PBS using a fixed tissue-to-buffer ratio of 1:10 (*w*/*v*; 100 mg tissue per 1 mL PBS) and further disrupted by brief sonication on ice (short pulses to avoid heating). Accordingly, the PBS volume was adjusted based on tissue weight (e.g., 300–500 mg tissue in 3–5 mL PBS). Homogenates were centrifuged at 1500× *g* for 15 min at 4 °C, and the supernatant was collected and immediately used for VEGF quantification. Because all specimens were processed using an identical tissue-to-buffer ratio (1:10, *w*/*v*) and identical centrifugation conditions, VEGF concentrations in homogenate supernatants are reported as pg/mL. We did not additionally normalize VEGF to total protein content, which should be considered in future studies. Samples were thawed only once for analysis to minimize freeze–thaw effects.

### 2.4. Characteristics of Enzyme-Linked Immunoassay Test

VEGF in tissue homogenate supernatants was quantified using a chemiluminescent sandwich ELISA as detailed below.

### 2.5. VEGF Test Kit

VEGF was quantified using the Human VEGF QuantiGlo ELISA Kit (QVE00B; R&D Systems, Minneapolis, MN, USA), which was designed to measure human VEGF_165_ (VEGF-A isoform).

Preparation of the assay in each well was carried out using the following steps:

(1) Assay Diluent RD1-8 (150 μL) was added;
(2) Standard or sample (50 μL) was added and incubated for 2 h at room temperature on a horizontal orbital microplate shaker set at 500 rpm;
(3) The liquid was aspirated and the wells were washed 3 times before filling each well with wash buffer (400 μL);
(4) After the last wash, any residual wash buffer was removed by decantation;
(5) Human conjugate (200 μL) was added to each well, which was covered with a new adhesive strip and incubated for 3 h at room temperature on a shaker;
(6) The liquid was aspirated and the wells were washed 4 times;
(7) Glo working reagent (100 μL) was added and incubated in the dark (at room temperature) for 5–20 min.
(8) After this time, the RLU of each well was determined using a luminometer set to the following parameters: 1.0 min delay time; 0.5 s/well reading time.

RLU was measured using an ELISA plate reader (Infinite^®^ 200 PRO, Tecan Group Ltd., Männedorf, Switzerland). A standard curve was generated by preparing 7 Human VEGF Standard mixtures: 0, 6.4, 32, 160, 800, 4000 and 20,000 pg/mL.

All standards and samples were measured in duplicate. Measurements were accepted if the intra-assay coefficient of variation (CV) between duplicates was ≤15%; otherwise, the sample was re-assayed.

### 2.6. Statistical Analysis

Due to the small sample size and the presence of potentially influential observations, VEGF concentrations were summarized primarily using the median and interquartile range (IQR). Between-group comparisons were performed using the Mann–Whitney U test. Effect size was reported as Cliff’s delta with 95% confidence intervals (CIs) estimated by bootstrap resampling. For transparency, a Welch’s *t*-test on untransformed values was additionally provided as a sensitivity analysis. Statistical significance was set at α = 0.05 (two-sided).

## 3. Results

### 3.1. VEGF Concentrations Show Inter-Individual Variability with Partial Overlap Between Tumor and Control Tissue

**Figure 1:**
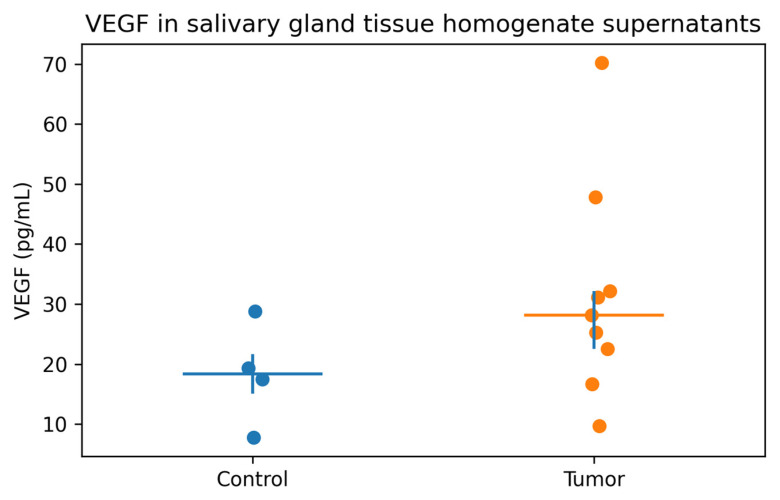
Individual VEGF concentrations in tissue homogenate supernatants from salivary gland tumors and control salivary gland tissues. Each dot represents one tissue sample (tumors, *n* = 9; controls, *n* = 4). Horizontal lines indicate the median and interquartile range (IQR). Due to the small cohort size and the presence of influential observations, group differences were assessed using robust summaries (median/IQR) and non-parametric testing; individual values are provided in Supplementary Table S1.

VEGF concentrations measured in tissue homogenate supernatants showed substantial inter-individual variability in both groups (Figure 1; Table 1). The tumor group displayed a higher central tendency than controls (median: 28.1 pg/mL vs. 18.4 pg/mL), although values partially overlapped. The between-group difference did not reach statistical significance in this exploratory cohort (Mann–Whitney U test, *p* = 0.199; Welch’s *t*-test as sensitivity analysis, *p* = 0.1024). Effect size estimates suggested a moderate-to-large magnitude (Hedges g = 0.767; Cliff’s delta = 0.50), but uncertainty remained considerable due to the small sample size. Bootstrap confidence intervals were wide, reflecting uncertainty in this small cohort.

### 3.2. Descriptive Assessment of Non-Oncological Control Samples

Because the control group consisted of only four non-oncological patients, a formal statistical subgroup analysis according to surgical indication was not performed. However, individual control values were descriptively reviewed in relation to the underlying non-neoplastic indications. This assessment did not allow us to identify a consistent pattern linking a specific benign condition with increased VEGF concentration. Nevertheless, the observed variability among control samples suggests that non-neoplastic processes may contribute to baseline VEGF levels in salivary gland tissue.

### 3.3. Sensitivity Analysis Excluding the Highest Tumor Value

One tumor sample exhibited a relatively high VEGF concentration (70.2 pg/mL), emphasizing biological variability and the need for cautious interpretation in a small exploratory cohort.

After excluding the highest tumor value (T2 = 70.2 pg/mL), the direction of the difference remained the same but was attenuated (Welch’s *p* = 0.1948; Mann–Whitney *p* = 0.2828), indicating that inferences in this pilot dataset are sensitive to influential observations and should be considered hypothesis-generating.

Overall, VEGF concentrations showed substantial inter-individual variability and partial overlap between tumor and control tissues. Although tumor samples showed a higher central tendency than controls, the difference did not reach statistical significance in this exploratory cohort. Sensitivity analysis excluding the highest tumor value showed an attenuation of the observed difference, confirming that the results are sensitive to influential observations and should be interpreted cautiously.

## 4. Discussion

**Figure 2:**
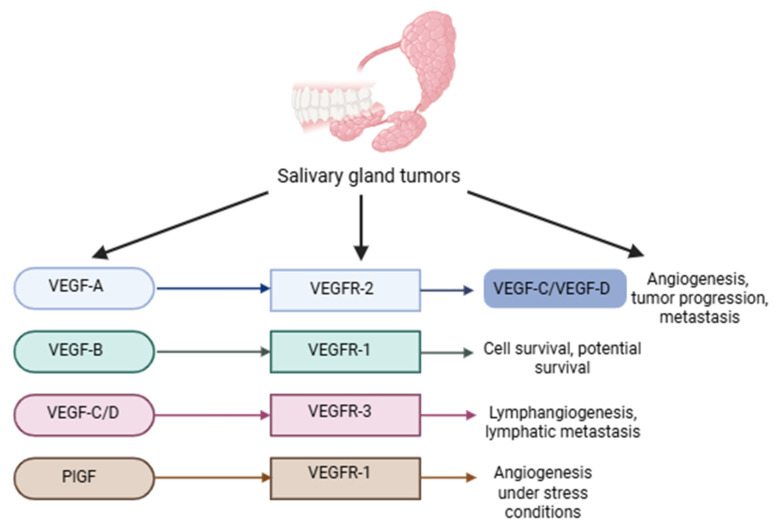
The role of VEGFs in salivary gland tumors. The schematic summarizes the involvement of VEGF-A, VEGF-B, VEGF-C, VEGF-D, and PlGF in angiogenesis, lymphangiogenesis, tumor progression, immune/microenvironmental regulation, and their potential relevance as biomarkers in salivary gland tumors.

The aim of this study was to evaluate differences in VEGF concentrations in salivary gland tissues affected by neoplastic changes and in macroscopically normal submandibular gland tissue obtained from patients who underwent surgery for non-neoplastic reasons. The hypothesis was that salivary gland tumor tissues are characterized by higher VEGF concentrations than tissues without neoplastic changes. The analysis showed that the mean VEGF concentration in tumor tissues was 31.5 ± 18.0 pg/mL, whereas in the control group, it was 18.3 ± 8.6 pg/mL. Despite a clear trend indicating higher VEGF levels in the tumor group, the statistical analysis did not find these differences to be statistically significant. At the same time, the results indicating higher VEGF concentrations in tumor tissues are consistent with current reports on the role of angiogenesis in salivary gland and head and neck cancers. Recent studies emphasize that VEGF overexpression correlates not only with tumor presence but also with its ability to invade and metastasize [16,17]. The upward trend observed in this study is thus consistent with this widely described biological mechanism. To better illustrate the biological context of these findings, Figure 2 summarizes the potential role of VEGF-related factors in salivary gland tumors, including angiogenesis, lymphangiogenesis, tumor microenvironment regulation, and as potential biomarkers.

It has been shown that VEGF is constitutively expressed in normal human salivary glands and secreted into saliva in healthy individuals. Consequently, baseline VEGF production in non-neoplastic tissue may contribute to interindividual variability and partial overlap of results between the tumor and control groups when measured in tissue homogenate supernatants [18,19]. Benign or non-neoplastic processes, particularly those associated with chronic inflammation, duct obstruction, tissue remodeling, ischemia, or local hypoxia, may stimulate angiogenic signaling and increase VEGF expression. Therefore, VEGF levels measured in macroscopically normal salivary gland tissue may not represent a completely “biologically inactive” baseline. This may partly explain the inter-individual variability and partial overlap between tumor and control samples observed in the present study. Due to the small number of control patients, this issue could only be assessed descriptively and should be investigated in larger cohorts with well-defined non-oncological control groups.

In this context, the obtained results should be interpreted as reflecting a complex microenvironmental signal rather than as an unambiguous diagnostic marker [20]. In the present study, VEGF was quantified in tissue homogenate supernatants using a chemiluminescent ELISA, which allows for quantitative measurement of soluble VEGF concentrations but does not provide information on the cellular source or spatial distribution of VEGF within the tissue. For example, Xu et al. reported that elevated serum VEGF levels may predict recurrence in patients with advanced-stage esophageal squamous cell carcinoma after curative esophagectomy followed by chemotherapy or concurrent radiotherapy [21]. Although these findings concern another cancer type, they support the broader prognostic relevance of circulating VEGF and highlight the need to interpret VEGF values in a tumor-specific context. Other complementary methods, such as mRNA expression analysis, multiplex protein assays, or digital image analysis of vascular markers, may further improve the biological interpretation of VEGF-related signaling. Therefore, combining quantitative assays with tissue-based methods such as immunohistochemistry may provide a more complete assessment of angiogenic activity in salivary gland tumors. Therefore, combining VEGF measurements with the analysis of receptors and co-receptors (e.g., VEGFR2/NRP1) or vascular markers may provide more information, allowing for a better assessment of angiogenic pathway activity than ligand concentration alone [20,21,22,23]. To summarize the broader biological relevance of VEGF-related factors in salivary gland tumors, Table 2 presents their main receptors, functional roles, and potential biomarker significance.

The lack of statistical significance despite a visible difference in medians may result from sample size limitations and high biological variability, which is frequently reported in recent translational studies. Analyses of angiogenesis biomarkers indicate that VEGF expression is characterized by significant heterogeneity both between patients and within the tumor itself [24]. Importantly, the current approach emphasizes the greater significance of effect size over the *p*-value itself in pilot studies, particularly with small cohorts. In this context, a moderate-to-large effect may indicate a genuine biological difference that requires confirmation.

From a mechanistic perspective, VEGF concentration in salivary gland tissues is the result of signals originating from various cell populations, including tumor cells, stromal fibroblasts, endothelial cells, and inflammatory infiltrates [25,26]. Hypoxia plays a key role in the activation of angiogenesis in solid tumors and can induce VEGF production in salivary gland cancer models, which is consistent with HIF-dependent activation of VEGF-A transcription [27,28,29,30]. In this context, VEGF can be considered not only as a marker of neovascularization but also as an indicator of microenvironmental stress and cellular metabolic adaptation [25,31]. The observed outlier value (70.2 pg/mL) may reflect differences in the tumor microenvironment, such as severe hypoxia, increased inflammatory cell activity, or a higher degree of malignancy. Published studies indicate that VEGF expression may be regulated by hypoxia-related mechanisms, particularly through HIF-1α-dependent signaling, and may increase in more aggressive tumor phenotypes [32,33]. For example, Zhou et al. showed that VEGF-A/C/D expression may be modulated through EGFR and HIF-1α signaling in lung adenocarcinoma, supporting the broader role of hypoxia-related pathways in angiogenesis and lymphangiogenesis [34]. The fact that the direction of the difference was maintained after removing the outlier suggests that the observed trend is stable. However, it also confirms that the results are highly sensitive to individual observations.

Recent publications indicate that VEGF is commonly overexpressed in salivary gland tumors and is associated with clinical and pathological features such as the Ki-67 proliferation index or tumor stage [35,36]. In a cohort study conducted in Vietnam involving 111 patients with salivary gland cancer, high VEGF expression was demonstrated immunohistochemically in approximately 66.7% of cases, suggesting its potential prognostic value and possible therapeutic target. High VEGF expression also correlated with proliferation markers such as Ki-67, highlighting the close links between angiogenesis and tumor aggressiveness [37].

Recent studies also focused on the interaction between angiogenesis and the immune microenvironment in salivary gland cancers. For example, immunophenotypic analysis revealed differences in epithelial–mesenchymal plasticity and in the profiles of vascular and immune cells between benign and malignant lesions, suggesting impaired control of angiogenesis and a potential mechanism promoting tumor progression [38].

Similar VEGFA-related signaling through PI3K/AKT and ERK pathways has also been described in other solid tumors, supporting the broader role of VEGF-associated pathways in tumor progression [39]. In addition to the classic VEGFR receptors, other molecules, such as CD73, can modulate the tumor microenvironment and the response to treatment, opening up new therapeutic perspectives in salivary gland cancers [38]. Comprehensive analysis of VEGF in combination with other microenvironmental markers may therefore enhance its prognostic value and identify potential therapeutic targets. Additionally, inflammatory pathways are closely linked to angiogenesis. NF-κB activation can induce iNOS and VEGF expression in salivary gland cancer cells and increase endothelial cell migration, supporting a model in which inflammation enhances the proangiogenic response [40,41,42]. Co-receptors such as neuropilins, which modulate VEGF–VEGFR signaling, have been linked to hematogenous metastasis in salivary gland cancer, indicating that the strength of the angiogenic signal depends not only on VEGF concentration but also on the receptor context [43,44,45]. These mechanisms may partially explain the observed interindividual variability and the overlap in results in tissue homogenate analyses [46].

The observed trend of elevated VEGF levels in patients with salivary gland cancer, despite the lack of statistical significance, may be of biological and clinical importance. VEGF is a key regulator of angiogenesis [47] and plays a fundamental role in the development and progression of cancers [48], including salivary gland cancers [49,50]. Elevated VEGF levels may reflect enhanced angiogenesis and vascular remodeling processes within the tumor [40,41,42,46,51,52,53,54,55]. Many studies have demonstrated a correlation between high VEGF levels and increased tumor aggressiveness, the risk of metastasis, and poorer prognosis [46,52,53,56,57,58,59,60,61,62,63,64]. However, it should be noted that this relationship may vary depending on the VEGF isoform and histological type of the tumor—some studies do not support this relationship [65,66,67,68], and some even indicate the opposite trend [69,70]. In the context of this study, the obtained results should be considered hypothesis-generating and point the way for future analyses involving larger cohorts and correlation with clinicopathological parameters.

From a clinical perspective, VEGF also remains a potential biomarker and therapeutic target in salivary gland cancers [13,15,71]. However, the obtained results should be considered hypothesis-generating and insufficient to draw diagnostic or prognostic conclusions in this small cohort. Clinical trials are currently underway for therapies targeting the VEGF/VEGFR pathway [72,73,74,75]. Further studies are needed, including larger patient groups and integrating VEGF assays with analyses of other components of the angiogenic pathway. The lack of statistical significance may be due to the limited sample size and the high heterogeneity of salivary gland tumors (differences in histological type, stage, or presence of perineural invasion), which were not accounted for in the analysis. In addition, VEGF assays of tissue homogenates reflect the combined signal from multiple cell types, which may increase the variability of the results.

The literature often demonstrates higher VEGF expression in malignant tumors compared to benign ones, but the results are heterogeneous and dependent on the histological type and study methodology [18,76,77,78]. This variability confirms that VEGF signaling is dependent on the biological context, and VEGF concentration alone may be insufficient to differentiate between neoplastic lesions. Furthermore, VEGF expression in benign tumors can vary significantly—from low to high [77,79,80,81,82,83]. The main limitation of the study is the small sample size, which affects the statistical power of the analysis. Potential confounding factors, such as patient clinical heterogeneity, age, smoking status, and inflammation, which may influence VEGF levels, should also be considered [84,85,86]. It should also be noted that the presence of different VEGF isoforms may complicate the interpretation of the results.

Future studies should include larger, clinically stratified cohorts representing different histological types of salivary gland tumors, such as mucoepidermoid carcinoma, adenoid cystic carcinoma, acinic cell carcinoma, salivary duct carcinoma, and carcinoma ex pleomorphic adenoma. In addition, future analyses should compare different methods of VEGF assessment, including ELISA-based quantification of tissue homogenates or blood samples, immunohistochemical evaluation of VEGF and VEGF receptors in tumor tissue, and molecular analyses of VEGF-related pathways, including HIF-1α, VEGFR2, VEGFR3, NRP1, and vascular markers such as CD31. Such an integrated approach may help determine whether VEGF has diagnostic, prognostic, or therapeutic relevance in specific salivary gland tumor subtypes.

## 5. Limitations

This study is limited by the small sample size, which restricts statistical power and increases sensitivity to influential observations. Another limitation is the small and clinically heterogeneous control group. Although control specimens were macroscopically normal and histopathologically free of neoplastic lesions, the underlying non-oncological conditions requiring surgery may have affected local VEGF expression. Inflammatory, obstructive, or remodeling-related processes could contribute to baseline VEGF variability in control tissue.

We did not normalize VEGF concentrations to total protein in the supernatant, which may contribute to variability across heterogeneous tissue matrices. Moreover, tissue homogenate supernatants represent a mixed biological signal derived from multiple cellular compartments, including tumor cells, stroma, endothelium, and immune cells. Therefore, VEGF concentration alone may not fully capture angiogenic pathway activity without accompanying receptor- and microenvironment-focused readouts.

## 6. Conclusions

In this exploratory pilot study, VEGF concentrations tended to be higher in salivary gland tumor tissues than in macroscopically normal control salivary gland tissues. However, the observed difference did not reach statistical significance, and partial overlap between groups was present. These findings suggest that VEGF may reflect angiogenic and microenvironmental activity in salivary gland tumors, but VEGF concentration alone is insufficient to draw diagnostic or prognostic conclusions in this small cohort.

Future studies should include larger, clinically stratified cohorts and integrate VEGF measurements with receptor expression, vascular markers, and clinicopathological parameters to better define the biological and potential clinical significance of VEGF signaling in salivary gland malignancies.

## Figures and Tables

**Table 1:** VEGF concentrations in salivary gland tissue homogenate supernatants.

Group	*n*	Median (IQR), pg/mL	Min–Max, pg/mL	Mean ± SD, pg/mL
Tumor tissue homogenate supernatant	9	28.1	9.6–70.2	31.5 ± 18.0
		(22.5–32.2)		
Control salivary gland tissue homogenate supernatant	4	18.4	7.8–28.8	18.3 ± 8.6
		(15.0–21.7)		

Data are summarized primarily as median (IQR) due to small sample size and potential non-normal distribution; individual values are provided in Supplementary Table S1.

**Table 2:** The role of VEGFs and PlGF in salivary gland tumors: expression, receptors, and biomarker potential.

VEGF	Receptor Expression	Site Role in Salivary	Gland Tumors	Biomarker Potential
VEGF-A	VEGFR-2	Serum, tumor	Stimulates angiogenesis, correlates with recurrence and metastasis	High for prognosis and treatment monitoring
VEGF-B	VEGFR-1	Cancer tissues	Supports cell survival, possible impact on tumor progression	Medium, further research needed
VEGF-C	VEGFR-3	Tumor tissue, lymph	Lymphangiogenesis, potential lymphatic metastasis	High, prognostic for metastasis
VEGF-D	VEGFR-3	Neoplastic tissues	Lymphangiogenesis, lymphatic metastasis	Medium, adjuvant
PlGF	VEGFR-1	Stress tissues, tumor	Angiogenesis in pathological conditions	Low/medium, supportive marker

## Data Availability

The original contributions presented in this study are included in the article/Supplementary Materials. Further inquiries can be directed to the corresponding authors.

## Supplementary Materials

The following supporting information can be downloaded at: https://www.mdpi.com/article/10.3390/jcm15124557/s1, Table S1: Clinicopathological characteristics of salivary gland tumor samples.

## References

[1] Tanzawa A., Saito K., Ota M., Takahashi K., Ohno I., Hanazawa T., Uzawa K., Takiguchi Y. (2024). Salivary Gland-Type Cancers: Cross-Organ Demographics of a Rare Cancer. Int. J. Clin. Oncol..

[2] Skálová A., Hyrcza M.D., Leivo I. (2022). Update from the 5th Edition of the World Health Organization Classification of Head and Neck Tumors: Salivary Glands. Head Neck Pathol..

[3] Swid M.A., Li L., Drahnak E.M., Idom H., Quinones W. (2023). Updated Salivary Gland Immunohistochemistry: A Review. Arch. Pathol. Lab. Med..

[4] Skalova A., Hyrcza M.D. (2023). Proceedings of the North American Society of Head and Neck Pathology Companion Meeting, New Orleans, LA, March 12, 2023: Classification of Salivary Gland Tumors: Remaining Controversial Issues?. Head Neck Pathol..

[5] Kang Y., Li H., Liu Y., Li Z. (2024). Regulation of VEGF-A expression and VEGF-A-targeted therapy in malignant tumors. J. Cancer Res. Clin. Oncol..

[6] Lee C., Kim M.J., Kumar A., Lee H.W., Yang Y., Kim Y. (2025). Vascular endothelial growth factor signaling in health and disease: From molecular mechanisms to therapeutic perspectives. Signal Transduct. Target. Ther..

[7] Zhang R., Yao Y., Gao H., Hu X. (2024). Mechanisms of angiogenesis in tumour. Front. Oncol..

[8] Failla C.M., Carbone M.L., Ramondino C., Bruni E., Orecchia A. (2025). Vascular Endothelial Growth Factor (VEGF) Family and the Immune System: Activators or Inhibitors?. Biomedicines.

[9] Kane K., Edwards D., Chen J. (2025). The influence of endothelial metabolic reprogramming on the tumor microenvironment. Oncogene.

[10] Yan T., Shi J. (2024). Angiogenesis and EMT regulators in the tumor microenvironment in lung cancer and immunotherapy. Front. Immunol..

[11] Tarallo V., Trotta S.M., Panico S., D’ORsi L., Mercadante G., Cicatiello V., De Falco S. (2024). PlGF and VEGF-A/PlGF Heterodimer are Crucial for Recruitment and Activation of Immune Cells During Choroid Neovascularization. Investig. Ophthalmol. Vis. Sci..

[12] Huang Z., Li L., Zhao X., Jin H., Shen M., Li B., Zeng Y., Zhang Q., Wang Q., Wang M. (2025). scRNA-seq reveals that VEGF signaling mediates the response to neoadjuvant anlotinib combined with PD-1 blockade therapy in non-small cell lung cancer. J. Transl. Med..

[13] Jiao J., Wu Y., Wu S., Jiang J. (2025). Enhancing Colorectal Cancer Treatment Through VEGF/VEGFR Inhibitors and Immunotherapy. Curr. Treat. Options Oncol..

[14] Park Y., Hong M.-S., Lee W.-H., Kim J.-G., Kim K. (2021). Highly Sensitive Electrochemical Aptasensor for Detecting the VEGF165 Tumor Marker with PANI/CNT Nanocomposites. Biosensors.

[15] Dos Santos E., Ramos J.-C., Normando A.-G., Leme A.-F. (2021). Prognostic Value of the Immunohistochemical Expression of Vascular Endothelial Growth Factors in Malignant Salivary Gland Neoplasms: A Systematic Review and Meta-Analysis. Med. Oral Patol. Oral Cir. Bucal.

[16] Hyytiäinen A., Wahbi W., Väyrynen O., Saarilahti K., Karihtala P., Salo T., Al-Samadi A. (2021). Angiogenesis Inhibitors for Head and Neck Squamous Cell Carcinoma Treatment: Is There Still Hope?. Front. Oncol..

[17] Szydłowski K., Puchalski M., Ołdziej S., Kasprzyk-Tryk A., Skorek A., Tretiakow D. (2024). The Impact of Inflammation on the Etiopathogenesis of Benign Salivary Gland Tumors: A Scoping Review. Int. J. Mol. Sci..

[18] Pammer J., Weninger W., Mildner M., Burian M., Wojta J., Tschachler E. (1998). Vascular Endothelial Growth Factor Is Constitutively Expressed in Normal Human Salivary Glands and Is Secreted in the Saliva of Healthy Individuals. J. Pathol..

[19] Taichman N.S., Cruchley A.T., Fletcher L.M., Hagi-Pavli E.P., Paleolog E.M., Abrams W.R., Booth V., Edwards R.M., Malamud D. (1998). Vascular endothelial growth factor in normal human salivary glands and saliva: A possible role in the maintenance of mucosal homeostasis. Lab. Investig..

[20] Pouloudi D., Sotiriadis A., Theodorakidou M., Sarantis P., Pergaris A., Karamouzis M.V., Theocharis S. (2020). The Impact of Angiogenesis in the Most Common Salivary Gland Malignant Tumors. Int. J. Mol. Sci..

[21] Xu H., Cao H., Zhang J., Jing C., Wang Z., Wu J., Du M., Xu X., Ma R. (2023). Serum VEGF levels as a predictor of recurrence in patients with advanced-stage esophageal squamous cell carcinoma following curative esophagectomy followed by chemotherapy or concurrent radiotherapy. Mol. Clin. Oncol..

[22] Yu F., Jiang X.Z., Chen W.T., Zhao Y.F., Zhou X.J. (2003). Microvessel density and expression of vascular endothelial growth factor in adenoid cystic carcinoma of salivary gland. Shanghai Kou Qiang Yi Xue.

[23] Gelfand M.V., Hagan N., Tata A., Oh W.J., Lacoste B., Kang K.T., Kopycinska J., Bischoff J., Wang J.H., Gu C. (2014). Neuropilin-1 functions as a VEGFR2 co-receptor to guide developmental angiogenesis independent of ligand binding. eLife.

[24] Bakshi H.A., Quinn G.A., Nasef M.M., Mishra V., Aljabali A.A.A., El-Tanani M., Serrano-Aroca Á., Da Silva M.W., McCarron P.A., Tambuwala M.M. (2022). Crocin Inhibits Angiogenesis and Metastasis in Colon Cancer via TNF-α/NF-kB/VEGF Pathways. Cells.

[25] Lugano R., Ramachandran M., Dimberg A. (2020). Tumor angiogenesis: Causes, consequences, challenges and opportunities. Cell. Mol. Life Sci..

[26] Leone P., Malerba E., Susca N., Favoino E., Perosa F., Brunori G., Prete M., Racanelli V. (2024). Endothelial cells in tumor microenvironment: Insights and perspectives. Front. Immunol..

[27] Hanahan D., Folkman J. (1996). Patterns and emerging mechanisms of the angiogenic switch during tumorigenesis. Cell.

[28] Maxwell P.H., Dachs G.U., Gleadle J.M., Nicholls L.G., Harris A.L., Stratford I.J., Hankinson O., Pugh C.W., Ratcliffe P.J. (1997). Hypoxia-inducible factor-1 modulates gene expression in solid tumors and influences both angiogenesis and tumor growth. Proc. Natl. Acad. Sci. USA.

[29] Kondo S., Mukudai Y., Soga D., Nishida T., Takigawa M., Shirota T. (2014). Differential expression of vascular endothelial growth factor in high- and low-metastasis cell lines of salivary gland adenoid cystic carcinoma. Anticancer Res..

[30] Forsythe J.A., Jiang B.H., Iyer N.V., Agani F., Leung S.W., Koos R.D., Semenza G.L. (1996). Activation of vascular endothelial growth factor gene transcription by hypoxia-inducible factor 1. Mol. Cell. Biol..

[31] Manalo D.J., Rowan A., Lavoie T., Natarajan L., Kelly B.D., Ye S.Q., Garcia J.G., Semenza G.L. (2005). Transcriptional regulation of vascular endothelial cell responses to hypoxia by HIF-1. Blood.

[32] Rankin E.B., Nam J.M., Giaccia A.J. (2016). Hypoxia: Signaling the Metastatic Cascade. Trends Cancer.

[33] Semenza G.L. (2002). HIF-1 and tumor progression: Pathophysiology and therapeutics. Trends Mol. Med..

[34] Zhou H., Geng F., Chen Y., Du J., Zhang X., Liu B., Song D., Hou H., Zhao H. (2021). The Mineral Dust-Induced Gene, Mdig, Regulates Angiogenesis and Lymphangiogenesis in Lung Adenocarcinoma by Modulating the Expression of VEGF-A/C/D via EGFR and HIF-1α Signaling. Oncol. Rep..

[35] Nguyen T.D., Nguyen H.T., Huynh C.G., Thai T.T., Bui T.X., Thai T.A., Duong T.T., Nguyen M.D., Vo D.A., Pham U.V. (2025). Expression of CD73 and VEGF in salivary gland carcinomas: Associations with clinicopathological characteristics in Vietnamese population. BMC Cancer.

[36] Ranjbar M.A., Ranjbar Z., Zahed M., Nikookar N. (2019). CD73 a novel marker for the diagnosis of benign and malignant salivary gland tumors. J. Clin. Exp. Dent..

[37] Sausa M., Vergilio G., Barone R., Porcasi R., Ofori P., Haddad F.A., Rappa F., Levi-Schaffer F., Leone A. (2025). Epithelial-Immune Cell Crosstalk in Salivary Gland Tumors: Implications for Tumor Progression and Diagnostic Assessment. Int. J. Mol. Sci..

[38] Allard B., Longhi M.S., Robson S.C., Stagg J. (2017). The ectonucleotidases CD39 and CD73: Novel checkpoint inhibitor targets. Immunol. Rev..

[39] Liu X., He H., Zhang F., Hu X., Bi F., Li K., Yu H., Zhao Y., Teng X., Li J. (2022). m6A Methylated EphA2 and VEGFA through IGF2BP2/3 Regulation Promotes Vasculogenic Mimicry in Colorectal Cancer via PI3K/AKT and ERK1/2 Signaling. Cell Death Dis..

[40] Zhang J., Peng B., Chen X. (2005). Expressions of Nuclear Factor kappaB, Inducible Nitric Oxide Synthase, and Vascular Endothelial Growth Factor in Adenoid Cystic Carcinoma of Salivary Glands: Correlations with the Angiogenesis and Clinical Outcome. Clin. Cancer Res..

[41] Zhang J., Peng B. (2007). In Vitro Angiogenesis and Expression of Nuclear Factor kappaB and VEGF in High and Low Metastasis Cell Lines of Salivary Gland Adenoid Cystic Carcinoma. BMC Cancer.

[42] Zhang J., Peng B. (2009). NF-kappaB Promotes iNOS and VEGF Expression in Salivary Gland Adenoid Cystic Carcinoma Cells and Enhances Endothelial Cell Motility in vitro. Cell Prolif..

[43] Soker S., Takashima S., Miao H.Q., Neufeld G., Klagsbrun M. (1998). Neuropilin-1 Is Expressed by Endothelial and Tumor Cells as an Isoform-Specific Receptor for Vascular Endothelial Growth Factor. Cell.

[44] Soker S., Miao H.Q., Nomi M., Takashima S., Klagsbrun M. (2002). VEGF165 mediates formation of complexes containing VEGFR-2 and neuropilin-1 that enhance VEGF165-receptor binding. J. Cell. Biochem..

[45] Djordjevic S., Driscoll P.C. (2013). Targeting VEGF signalling via the neuropilin co-receptor. Drug Discov. Today.

[46] Ni Q., Sun J., Ma C., Li Y., Ju J., Sun M. (2018). The Neuropilins and Their Ligands in Hematogenous Metastasis of Salivary Adenoid Cystic Carcinoma-An Immunohistochemical Study. J. Oral Maxillofac. Surg..

[47] Melincovici C.S., Boşca A.B., Şuşman S., Mărginean M., Mihu C., Istrate M., Moldovan I.M., Roman A.L., Mihu C.M. (2018). Vascular Endothelial Growth Factor (VEGF)—Key Factor in Normal and Pathological Angiogenesis. Rom. J. Morphol. Embryol..

[48] Carmeliet P. (2005). VEGF as a Key Mediator of Angiogenesis in Cancer. Oncology.

[49] Ishibashi H., Shiratuchi T., Nakagawa K., Onimaru M., Sugiura T., Sueishi K., Shirasuna K. (2001). Hypoxia-Induced Angiogenesis of Cultured Human Salivary Gland Carcinoma Cells Enhances Vascular Endothelial Growth Factor Production and Basic Fibroblast Growth Factor Release. Oral Oncol..

[50] Kim J.W., Kwon G.Y., Roh J.-L., Choi S.-H., Nam S.Y., Kim S.Y., Cho K.-J. (2011). Carcinoma Ex Pleomorphic Adenoma of the Salivary Glands: Distinct Clinicopathologic Features and Immunoprofiles between Subgroups According to Cellular Differentiation. J. Korean Med. Sci..

[51] Park Y., In Y. (2006). Immunohistochemical assays for the expression of angiogenic signaling molecules and microvessel density in adenoid cystic carcinomas of human salivary glands. J. Korean Assoc. Oral Maxillofac. Surg..

[52] Doi R., Kuratate I., Okamoto E., Ryoke K., Ito H. (1999). Expression of P53 Oncoprotein Increases Intratumoral Microvessel Formation in Human Salivary Gland Carcinomas. J. Oral Pathol. Med..

[53] Shi L., Chen X.-M., Wang L., Zhang L., Chen Z. (2007). Expression of Caveolin-1 in Mucoepidermoid Carcinoma of the Salivary Glands: Correlation with Vascular Endothelial Growth Factor, Microvessel Density, and Clinical Outcome. Cancer.

[54] Shieh Y.-S., Hung Y.-J., Hsieh C.-B., Chen J.-S., Chou K.-C., Liu S.-Y. (2009). Tumor-Associated Macrophage Correlated with Angiogenesis and Progression of Mucoepidermoid Carcinoma of Salivary Glands. Ann. Surg. Oncol..

[55] Wang H.-F., Wang S.-S., Zheng M., Dai L.-L., Wang K., Gao X.-L., Cao M.-X., Yu X.-H., Pang X., Zhang M. (2019). Hypoxia Promotes Vasculogenic Mimicry Formation by Vascular Endothelial Growth Factor A Mediating Epithelial-Mesenchymal Transition in Salivary Adenoid Cystic Carcinoma. Cell Prolif..

[56] Hao L., Xiao-lin N., Qi C., Yi-ping Y., Jia-quan L., Yan-ning L. (2010). Nerve Growth Factor and Vascular Endothelial Growth Factor: Retrospective Analysis of 63 Patients with Salivary Adenoid Cystic Carcinoma. Int. J. Oral Sci..

[57] Liu H., Chen L., Wang C., Zhou H. (2022). The Expression and Significance of Vascular Endothelial Growth Factor A in Adenoid Cystic Carcinoma of Palatal Salivary Gland. Eur. Arch. Otorhinolaryngol..

[58] Ou Yang K., Liang J., Huang Z. (2011). Association of Clinicopathologic Parameters with the Expression of Inducible Nitric Oxide Synthase and Vascular Endothelial Growth Factor in Mucoepidermoid Carcinoma. Oral Dis..

[59] Lequerica-Fernández P., Astudillo A., De Vicente J.C. (2007). Expression of Vascular Endothelial Growth Factor in Salivary Gland Carcinomas Correlates with Lymph Node Metastasis. Anticancer Res..

[60] Lim J., Kang S., Lee M., Pai H., Yoon H., Lee J., Hong S., Lim C. (2003). Expression of Vascular Endothelial Growth Factor in Salivary Gland Carcinomas and Its Relation to P53, Ki-67 and Prognosis. J. Oral Pathol. Med..

[61] Liu H., Chen L., Wang C., Zhou H. (2023). Matrix Metalloproteinase 7 Is Associated with Clinical and Pathological Characteristics of Salivary Adenoid Cystic Carcinomas. Eur. Arch. Otorhinolaryngol..

[62] Mariam M.S., Syawqie A., Cahyanto A. (2020). Immunoexpression of E-Cadherin and VEGF-A Proteins in Various Degrees of Histologic Malignancies of Adenoid Cystic Carcinoma of Salivary Glands. J. Int. Dent. Med. Res..

[63] Park S., Nam S., Keam B., Kim T., Jeon Y., Lee S., Hah J., Kwon T., Kim D., Sung M. (2016). VEGF and Ki-67 Overexpression in Predicting Poor Overall Survival in Adenoid Cystic Carcinoma. Cancer Res. Treat..

[64] Stárek I., Salzman R., Kučerová L., Skálová A., Hauer L. (2015). Expression of VEGF-C/-D and Lymphangiogenesis in Salivary Adenoid Cystic Carcinoma. Pathol. Res. Pract..

[65] Mello M.F., Costa A.F., Freitas L.L., Soares A.B., Araujo V.C., Tincani A.J., Martins A.S., Altemani A. (2011). Lymphatic Vessel Density and Expressions of Lymphangiogenic Growth Factors in Salivary Carcinomas. Neoplasma.

[66] Shamloo N., Taghavi N., Yazdani F., Azimian P., Ahmadi S. (2020). Evaluation of VEGF Expression Correlates with COX-2 Expression in Pleomorphic Adenoma, Mucoepidermoid Carcinoma and Adenoid Cystic Carcinoma. Dent. Res. J..

[67] Lee S.K., Kwon M.S., Lee Y.S., Choi S.-H., Kim S.Y., Cho K.J., Nam S.Y. (2012). Prognostic Value of Expression of Molecular Markers in Adenoid Cystic Cancer of the Salivary Glands Compared with Lymph Node Metastasis: A Retrospective Study. World J. Surg. Oncol..

[68] Ko Y.H., Roh J.H., Son Y.-I., Chung M.K., Jang J.Y., Byun H., Baek C.-H., Jeong H.-S. (2010). Expression of Mitotic Checkpoint Proteins BUB1B and MAD2L1 in Salivary Duct Carcinomas. J. Oral Pathol. Med..

[69] Gleber-Netto F.O., Florêncio T.N.G., de Sousa S.F., Abreu M.H.N.G., Mendonça E.F., Aguiar M.C.F. (2012). Angiogenesis and Lymphangiogenesis in Mucoepidermoid Carcinoma of Minor Salivary Glands. J. Oral Pathol. Med..

[70] Zhang Y., Brekken R.A. (2022). Direct and indirect regulation of the tumor immune microenvironment by VEGF. J. Leukoc. Biol..

[71] Adwani A., Kheur S., Kheur M., Mahajan P. (2021). Prognostic Biomarkers for Salivary Adenoid Cystic Carcinoma: A Systematic Review. Clin. Cancer Investig. J..

[72] Agulnik M., Siu L.L. (2004). An Update on the Systemic Therapy of Malignant Salivary Gland Cancers: Role of Chemotherapy and Molecular Targeted Agents. Curr. Med. Chem. Anticancer Agents.

[73] Chau N.G., Hotte S.J., Chen E.X., Chin S.F., Turner S., Wang L., Siu L.L. (2012). A Phase II Study of Sunitinib in Recurrent and/or Metastatic Adenoid Cystic Carcinoma (ACC) of the Salivary Glands: Current Progress and Challenges in Evaluating Molecularly Targeted Agents in ACC. Ann. Oncol..

[74] Hanna G.J., Ahn M.-J., Muzaffar J., Keam B., Bowles D.W., Wong D.J., Ho A.L., Kim S.-B., Worden F., Yun T. (2023). A Phase II Trial of Rivoceranib, an Oral Vascular Endothelial Growth Factor Receptor 2 Inhibitor, for Recurrent or Metastatic Adenoid Cystic Carcinoma. Clin. Cancer Res..

[75] Park Y., Kang H., Park J. (2008). Antivascular Therapy via Inhibition of Receptor Tyrosine Kinases in an Orthotopic Murine Model of Salivary Adenoid Cystic Carcinoma. J. Korean Assoc. Oral Maxillofac. Surg..

[76] Younes M.N., Park Y.W., Yazici Y.D., Gu M., Santillan A.A., Nong X., Kim S., Jasser S.A., El-Naggar A.K., Myers J.N. (2006). Concomitant Inhibition of Epidermal Growth Factor and Vascular Endothelial Growth Factor Receptor Tyrosine Kinases Reduces Growth and Metastasis of Human Salivary Adenoid Cystic Carcinoma in an Orthotopic Nude Mouse Model. Mol. Cancer Ther..

[77] Faur A.C., Lazar E., Cornianu M. (2014). Vascular Endothelial Growth Factor (VEGF) Expression and Microvascular Density in Salivary Gland Tumors. APMIS.

[78] de Faria P.R., Lima R.A., Dias F.L., de Faria P.A.S., Eisenberg A.L.A., do Nascimento Souza K.C., Cardoso S.V., Loyola A.M. (2011). Vascular Endothelial Growth Factor and Thymidine Phosphorylase Expression in Salivary Gland Tumors with Distinct Metastatic Behavior. J. Oral Pathol. Med..

[79] Fonseca F.P., Basso M.P.M., Mariano F.V., Kowalski L.P., Lopes M.A., Martins M.D., Rangel A.L.C.A., Santos-Silva A.R., Vargas P.A. (2015). Vascular Endothelial Growth Factor Immunoexpression Is Increased in Malignant Salivary Gland Tumors. Ann. Diagn. Pathol..

[80] Salzman R., Stárek I., Kučerová L., Skálová A., Hoza J. (2014). Neither Expression of VEGF-C/D nor Lymph Vessel Density Supports Lymphatic Invasion as the Mechanism Responsible for Local Spread of Recurrent Salivary Pleomorphic Adenoma. Virchows Arch..

[81] Soares A.B., Altemani A., de Oliveira T.R., de Oliveira Fonseca Rodrigues F., Ribeiro-Silva A., Soave D.F., Passador-Santos F., Brum S.T., Napimoga M.H., de Araújo V.C. (2015). Comparison of the Blood and Lymphatic Microvessel Density of Pleomorphic Adenoma and Basal Cell Adenoma. Clin. Med. Insights Pathol..

[82] Faur A., Gurban C., Cornianu M., Bolintineanu S., Tuta-Sas I., Stef D., Heredea R., Balabuc C. (2019). Vascular Morphogenesis in Warthin’s Tumor and Insights into Its Origin. Rom. Biotechnol. Lett..

[83] Swelam W., Ida-Yonemochi H., Maruyama S., Ohshiro K., Cheng J., Saku T. (2005). Vascular Endothelial Growth Factor in Salivary Pleomorphic Adenomas: One of the Reasons for Their Poorly Vascularized Stroma. Virchows Arch..

[84] Okamoto Y., Nagai T., Nakajo I., Seta K., Gotoh Y., Fujita N., Fukui T., Masuzawa T. (2008). Determination of Age-Related Changes in Human Vascular Endothelial Growth Factor in the Serum and Urine of Healthy Subjects. Clin. Lab..

[85] Ugur M.G., Kutlu R., Kilinc I. (2018). The Effects of Smoking on Vascular Endothelial Growth Factor and Inflammation Markers: A Case-Control Study. Clin. Respir. J..

[86] Sakellariou G.T., Iliopoulos A., Konsta M., Kenanidis E., Potoupnis M., Tsiridis E., Gavana E., Sayegh F.E. (2017). Serum Levels of Dkk-1, Sclerostin and VEGF in Patients with Ankylosing Spondylitis and Their Association with Smoking, and Clinical, Inflammatory and Radiographic Parameters. Jt. Bone Spine.

