# Supplementary material for: Tissue Homogenate VEGF Levels in Malignant Salivary Gland Tumors vs. Controls: An Exploratory Pilot Study
Source: J Clin Med. 2026 Jun 12;15(12):4557. doi: 10.3390/jcm15124557 (PMC13301534; doi:10.3390/jcm15124557)
Supplement: Supplementary file 1 [file jcm-15-04557-s001.zip › jcm-4304968-supplementary.pdf]

Table S1. Clinicopathological characteristics of salivary gland tumor samples.

Sample	Histology	Grade	Stage	Location
1	Mucoepidermoid carcinoma	High	III	Parotid
2	Adenoid cystic carcinoma	II	II	Submandibular
3	Acinic cell carcinoma	High-grade transformation	III	Parotid
4	Mucoepidermoid carcinoma	Intermediate	III	Parotid
5	Acinic cell carcinoma	High-grade transformation	II	Parotid
6	Mucoepidermoid carcinoma	High	III	Parotid
7	Salivary duct carcinoma	High	III	Parotid
8	Salivary duct carcinoma	High	III	Parotid
9	Salivary duct carcinoma	High	II	Parotid
